# Two minimally invasive fusion techniques for neurogenic claudication caused by degenerative lumbar spondylolisthesis: a minimum 2-year follow-up study

**DOI:** 10.3389/fneur.2025.1705975

**Published:** 2025-10-29

**Authors:** Wei Cui, Yehui Wang, Wei Hou, Xuangeng Deng

**Affiliations:** Spine & Neurosurgery Department, Sichuan Provincial Orthopaedic Hospital, Chengdu, China

**Keywords:** neurogenic claudication, degenerative lumbar spondylolisthesis, surgical technique, oblique lumbar interbody fusion, transforaminal lumbar interbody fusion

## Abstract

**Background:**

Neurogenic claudication induced by degenerative lumbar spondylolisthesis (DLS) is a highly prevalent condition. In recent research, oblique lumbar interbody fusion with anterior fixation (OLIF-AF) has emerged as a favored minimally invasive approach for treating DLS. Nevertheless, there have been relatively few investigations that have compared this method with minimally invasive transforaminal lumbar interbody fusion (MIS-TLIF), which has long been considered the standard technique.

**Aim:**

To compare the clinical and radiological outcomes of OLIF-AF and MIS-TLIF in patients suffering from single-level, low-grade degenerative lumbar spondylolisthesis that leads to neurogenic claudication.

**Methods:**

We conducted a retrospective study of 57 patients who presented with neurogenic claudication secondary to single-level degenerative lumbar spondylolisthesis and underwent surgical treatment between May 2018 and December 2022. Of these 57 patients, 31 underwent oblique lumbar interbody fusion with anterior fixation (OLIF-AF) and 26 underwent minimally invasive transforaminal lumbar interbody fusion (MIS-TLIF). Every patient had a follow-up period of at least 2 years. The recorded and compared data included the perioperative indicators, follow-up outcomes, and imaging parameters between the two groups.

**Results:**

Preoperatively, the two groups exhibited a comparable baseline in demographic data and clinical characteristics, including visual analog scale (VAS) scores, Oswestry Disability Index (ODI), disc height (DH), intervertebral space angle (ISA), spinal canal cross-sectional area (CSA) and slip percentage (SP). Postoperatively, both groups exhibited significant improvements in VAS and ODI scores. The OLIF-AF group demonstrated superior clinical outcomes in terms of operative time (125.7 ± 46.2 min vs. 202.1 ± 66.4 min, *p* < 0.001), estimated blood loss (58.6 ± 30.5 mL vs. 143.5 ± 46.8 mL, *p* < 0.001), and length of hospital stay (8.6 ± 2.5 days vs. 10.7 ± 3.5 days, *p* = 0.009) compared to the MIS-TLIF group. However, the incidence of perioperative complications did not differ significantly between the two groups (16.1% vs. 19.2%, *p* > 0.05). Radiographic assessment at the 2-year follow-up revealed significantly greater improvements in DH, ISA and CSA in the OLIF-AF group (*p* < 0.05). At the 2-year follow-up, there were no significant differences between the two groups in SP (9.6 ± 1.8 % vs. 9.4 ± 1.6 %), interbody fusion rate (93.5% vs. 92.3%), or cage subsidence rate (3.2% vs. 3.8%) (all *p* > 0.05). Although low back pain VAS, leg pain VAS, and ODI scores improved postoperatively in both groups compared with preoperative values, the OLIF-AF group showed greater improvement in low back pain VAS and ODI scores at 1 week and 3 months postoperatively.

**Conclusion:**

Both OLIF-AF and MIS-TLIF are efficient in treating neurogenic claudication resulting from degenerative lumbar spondylolisthesis (DLS). Nevertheless, OLIF-AF is associated with a shorter operation duration, reduced surgical trauma, and faster early recovery, while maintaining long-term effectiveness and safety comparable to those of MIS-TLIF.

## Introduction

Neurogenic claudication has emerged as a rapidly escalating public health challenge that disproportionately affects the expanding older-adult population. It manifests as unilateral or bilateral buttock pain and/or lower-limb discomfort, pain, weakness, or a sense of heaviness that is precipitated or exacerbated by standing or walking (1). The most common cause of neurogenic claudication is lumbar spinal stenosis, with approximately one-third of these cases resulting from low-grade degenerative lumbar spondylolisthesis (DLS) (2). DLS is commonly classified using the Meyerding classification, with grades I–II representing low-grade slips, which are the most prevalent form (3). Surgical intervention is indicated when conservative measures fail or when neurogenic claudication significantly impairs quality of life (4). Notably, 80% of surgical patients prefer minimally invasive surgical treatment (5).

Minimally invasive transforaminal lumbar interbody fusion (MIS-TLIF), introduced by Foley in 2002 (6), is characterized by less blood loss and faster recovery. It is considered the “gold standard” among minimally invasive fusion techniques for lumbar conditions (7). However, this approach requires manipulation of intraspinal neural structures and sustained retraction of paraspinal muscles, which can lead to nerve injury and persistent postoperative back pain (8). In recent years, oblique lumbar interbody fusion (OLIF) has attracted widespread attention. This technique accesses the disc space for fusion through the natural anatomical corridor between the abdominal great vessels and the psoas muscle, thereby restoring lumbar alignment and expanding the stenotic spinal canal (9). By avoiding both intraspinal neural manipulation and paraspinal muscle disruption, OLIF achieves excellent clinical outcomes (10). However, standalone OLIF for low-grade DLS carries a high risk of construct failure (11). Consequently, many surgeons combine OLIF with anterior single-screw-rod fixation (OLIF-AF) to enhance clinical efficacy for degenerative lumbar conditions (12, 13). Nevertheless, comparative studies evaluating whether OLIF-AF offers equivalent or superior clinical outcomes compared to the classic MIS-TLIF for degenerative spondylolisthesis are scarce.

This study seeks to evaluate and contrast the therapeutic effectiveness, radiographic findings, and safety profiles between MIS-TLIF and OLIF-AF for managing single-level DLS accompanied by neurogenic claudication, based on a minimum two-year follow-up period.

## Materials and methods

### General patient information

The Sichuan Provincial Orthopaedic Hospital Ethics Committee provided ethical approval (NO. KY2025-027-01). This retrospective analysis reviewed surgical records of patients treated with minimally invasive procedures for DLS accompanied by neurogenic claudication at an academic medical center from May 2018 to December 2022. Owing to the retrospective design, written informed consent was formally waived. Data for OLIF-AF were collected from May 2018 to December 2022, while data for MIS-TLIF were obtained from October 2018 to May 2022, during which the OLIF technique was applied for lumbar degenerative disease. Inclusion criteria were as follows: (1) persistent severe neurogenic claudication despite a minimum of 6 months of non-surgical management; (2) who were at least 18 years of age; (3) single-level degenerative lumbar spondylolisthesis; (4) Meyerding grade I–II on preoperative standing lateral X-ray of the lumbar spine; (5) minimum follow-up of 2 years. Exclusion criteria were as follows: (1) presence of a contraindication to anesthesia or surgery; (2) evident disc herniation or bony spinal stenosis within the lumbar canal; (3) L5 Spondylolisthesis, this is because the classic OLIF approach is designed for the L2-L5 levels, not for the L5-S1 level; (4) osteoporosis (hip bone mineral density T-score <−2.5SD); (5) history of spinal tumor, infection, or previous spinal surgery; (6) follow-up period shorter than 2 years. In total, we included 57 patients (corresponding to 57 surgical segments) in the present study. 31 patients received OLIF-AF, while 26 patients received MIS-TLIF. All of these operations were performed by one particular spinal surgery team.

### Surgical techniques

#### OLIF-AF

**Figure 1:**
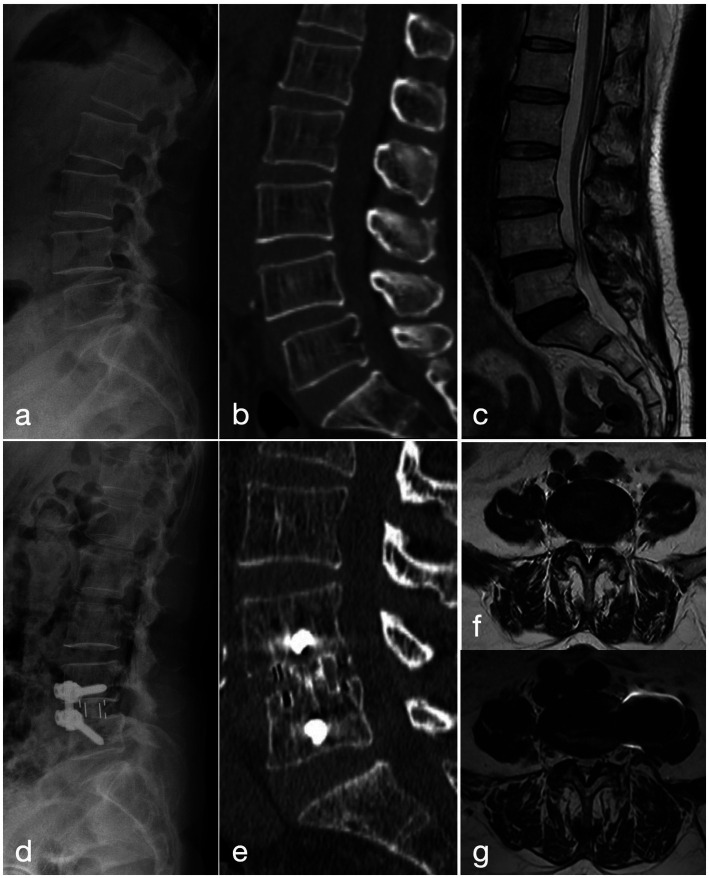
A 56-year-old woman who had been suffering from neurogenic claudication for 1 year, with symptoms worsening over the last 3 months, was treated with the OLIF-AF procedure. **(a–c)** Pre-operative lateral radiograph, CT and MRI show grade-I anterior spondylolisthesis of L4. **(d)** Post-operative lateral radiograph demonstrates well-positioned instrumentation and interbody cage with good reduction of the slip. **(e)** CT at 2 years confirms solid interbody fusion. **(f)** Pre-operative MRI reveals severe L4/5 central canal stenosis. **(g)** MRI at 2 years shows increased canal volume and positive nerve-root sedimentation sign.

The patient was placed in the right lateral decubitus position, with the operating table adjusted to induce a slight left-sided convexity of the lumbar spine. The target level was localized under C-arm fluoroscopy. After standard sterile preparation and draping, make a 5-cm oblique incision in the left lower quadrant. The external oblique aponeurosis was sharply incised, and the internal oblique, transversus abdominis, and transversalis fascia were bluntly split. The retroperitoneal space was entered by gently retracting the extraperitoneal fat, exposing the psoas muscle. A working corridor was created between the psoas and the great vessels/peritoneal structures, with constant care to protect the lumbar plexus and ureter. Under fluoroscopic control the target disc was incised under direct vision; the nucleus and cartilaginous endplates were removed and the space prepared. An allograft bone graft (Shanxi Aorui, China) was packed into an appropriately sized cage (Medtronic, USA) and inserted into the disc space under fluoroscopy. To achieve optimal reduction, we performed sequential clockwise reaming of the endplates. This was followed by a thorough release of the disc space and the implantation of a large cage to restore intervertebral height, lordotic angle, and overall alignment. After interbody placement, anterior single-screw-rod fixation was applied to the vertebral bodies immediately above and below the disc. Hemostasis was achieved, the wound was closed in layers, and no drain was placed (Figure 1).

#### MIS-TLIF

**Figure 2:**
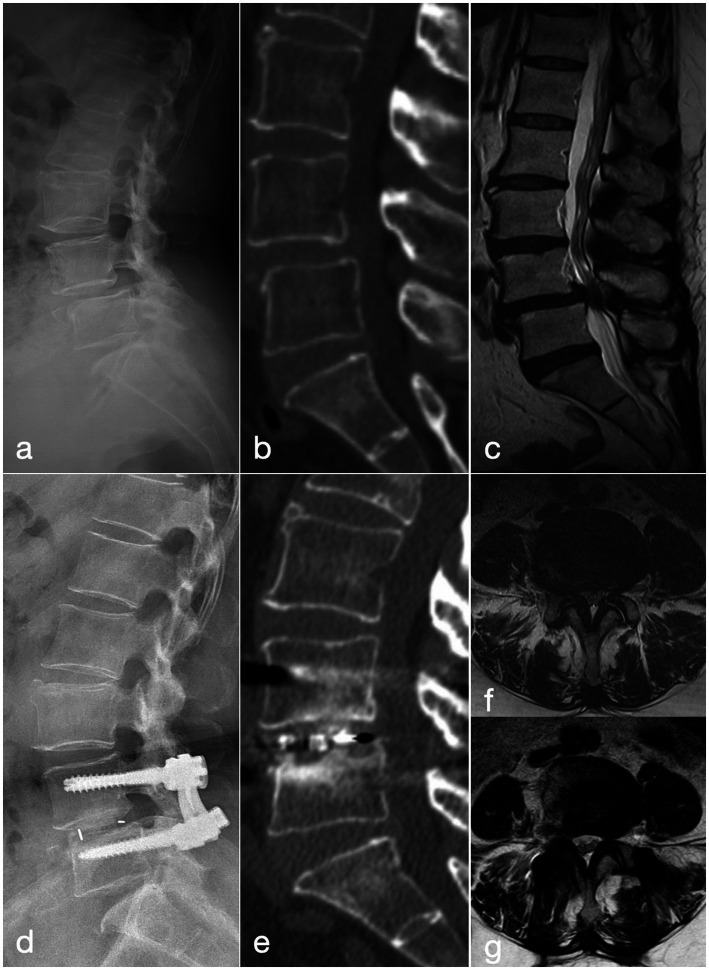
A 57-year-old woman with recurrent low back pain and lower limb intermittent claudication of 9 months’ duration, was treated with the MIS-TLIF procedure. **(a–c)** Pre-operative lateral radiograph, CT and MRI show grade-I anterior spondylolisthesis of L4. **(d)** Post-operative lateral radiograph demonstrates well-positioned instrumentation and interbody cage with good reduction of the slip. **(e)** CT at 2 years confirms solid interbody fusion at L4/5. **(f)** Pre-operative MRI reveals severe L4/5 central canal stenosis. **(g)** MRI at 2 years shows increased canal volume and patent cerebrospinal fluid signal.

The patient was positioned in a prone stance. The level of interest was identified with the assistance of C-arm fluoroscopy. An incision on the skin was made along the line that links the projection points of the lateral pedicle borders of the vertebrae above and below the targeted level. The subcutaneous tissues and skin were incised one after another. The multifidus and longissimus muscles were separated in a blunt manner to reach the facet joint of the relevant segment. A series of sequential dilators along with a tubular retractor were put in place. The superior and inferior articular processes and lamina were partially excised. A partial removal of the flaval ligament was carried out to uncover the lateral edge of the dura mater and nerve root. These were carefully retracted medially so as to make the disc visible. The material of the disc was taken out, and the endplates were made ready. An appropriately sized cage (Fule, China) was filled with the autologous bone graft that was acquired from the excised lamina and facet joints. Under fluoroscopic guidance, the cage was subsequently inserted into the disc space. After that, a bilateral Wiltse intermuscular approach was employed to reveal the facet joints and accessory processes. Under the guidance of fluoroscopy, pedicle screw systems were implanted at suitable levels. The rods were linked up, the reduction was achieved via compression or distraction operations, and the structures were fastened. During screw placement, an appropriate height difference should be maintained between the screws of the slipped vertebra and the inferior vertebra to facilitate subsequent reduction by lifting. During reduction, the rod-screw system is first locked onto the screws of the non-slipped inferior vertebra. Then, using the pre-set height difference between the screws, the slipped superior vertebra is gradually lifted to achieve reduction. Finally, the rod-screw system was secured. After achieving complete hemostasis, the wound was sutured in layers without placing a drain (Figure 2).

### Postoperative management

All patients received standard supportive care within 24 h after surgery, including antibiotics for infection prophylaxis, analgesia, and fluid infusion. Patients were allowed to ambulate with a brace when their VAS score for low back and leg pain was ≤ 4. The lumbar brace was strictly worn for 3 months postoperatively.

### The collection of demographic and perioperative data

Patient demographic and perioperative data were retrospectively reviewed. Demographic variables included age, sex, body mass index, symptom duration, length of hospital stay, and length of follow-up. Perioperative outcomes included surgical segment, Meyerding classification, intraoperative surgical time, estimated blood loss, and any intra- or postoperative complications occurring within 2 years. The health cost after medical insurance payment was also recorded.

### Clinical and radiographic outcomes

Assessment of Oswestry Disability Index (ODI) and visual analog scale (VAS) scores for low back and leg pain was performed preoperatively and postoperatively (at 1 week, 3 months, and 2 years). These scores were calculated and analyzed at each corresponding time point.

The radiographic evaluation included:

**Disc Height (DH):** The vertical distance between the midpoints of the superior and inferior endplates of the operated segment was measured as the disc height on lateral lumbar radiographs (14).

**Intervertebral Space Angle (ISA):** When looking at lateral lumbar radiographs, the segmental lordosis was gaged as the angle formed between the endplates of the disc space where the operation was performed.

**Spinal Canal Cross-Sectional Area (CSA):** On T2-weighted MRI, the spinal-canal contour at the mid-disc plane of the operated segment was manually delineated pre-operatively and at the 2-year follow-up; the enclosed area was measured to evaluate the extent of decompression (14).

**Slip Percentage (SP):** The percentage of the distance the superior vertebral body has slipped relative to the inferior vertebral body, measured against the length of the superior endplate of the inferior vertebral body.

**Fusion Rate:** Assessed using CT 3D reconstructions according to the Bridwell grading system. Grades I and II were considered successful fusion. (Grade I: trabecular bone bridging the endplates with intact implant; Grade II: graft not fully consolidated but intact implant with no lucency; Grade III: implant intact but definite lucency at implant-endplate interface with insufficient trabecular connection; Grade IV: definite subsidence or failure of fusion with resorption) (15).

**Cage Subsidence:** Diagnosed if the postoperative disc height decreased by more than 2 mm compared to the immediate postoperative measurement (16).

The radiographic assessments were conducted by senior attending spine surgeons blinded to the patient group.

### Statistical analysis

SPSS (IBM, Version 26.0) was employed to conduct all statistical analyses. Continuous variables that follow a normal distribution are presented as the mean ± standard deviation. For two - group analyses, intergroup comparisons were carried out by utilizing independent samples *t*-tests. To compare the variance of continuous numerical variables among groups, the one-way analysis of variance was utilized. The chi-square test or Fisher exact test was utilized to assess categorical data. Statistical significance was considered when the *p*-value was less than 0.05.

## Results

The baseline features of the study subjects are shown in Table 1. There were no statistically significant differences between the two groups in terms of age, gender, body mass index, surgical segment, Meyerding classification, or follow-up duration (*p* > 0.05).

**Table 1:** Patient demographic data.

Variables	OLIF-AF (*n* = 31)	MIS-TLIF (*n* = 26)	*p*-value
Age (year)	56.74 ± 11.22	56.35 ± 9.48	0.880
Sex			
Female	20	18	0.631
Male	11	8	
BMI (kg/m^2^)	25.99 ± 3.57	25.10 ± 3.67	0.351
Duration of disease (month)	6.9 ± 1.7	7.1 ± 1.4	0.375
Surgical segment			
L2-3	1	0	0.504
L3-4	2	1	
L4-5	28	25	
Meyerding classification			
I	28	24	0.960
II	3	2	
Follow-up (month)	26.9 ± 10.1	27.1 ± 9.8	0.622

Values are presented as mean ± standard deviation. BMI, body mass index.

### Perioperative outcomes

The mean operative time, estimated blood loss, and length of hospital stay in the MIS-TLIF group were significantly higher than those in the OLIF-AF group (202.1 ± 66.4 min vs. 125.7 ± 46.2 min, *p* < 0.001; 143.5 ± 46.8 mL vs. 58.6 ± 30.5 mL, *p* < 0.001; 10.7 ± 3.5 days vs. 8.6 ± 2.5 days, *p* = 0.009, respectively, Table 2). In the OLIF-AF group, perioperative complications included one case of hip flexor weakness and two cases of sympathetic chain injury, all of which recovered within 1 month after neurotrophic medication. Additionally, two cases of poor wound healing were observed and successfully managed with dressing changes. The overall complication rate was 16.1% (5/31). In the MIS-TLIF group, there was one case of worsened numbness in the dermatome corresponding to the surgical nerve root and one case of muscle weakness; both patients recovered within 1 month with neurotrophic treatment. Two cases of dural tears occurred during surgery, which were managed by covering the tear site with gelatin sponge before closure and performing meticulous suturing of the deep fascia. Postoperatively, a Trendelenburg position was adopted to reduce intrathecal pressure. One case of poor wound healing was treated successfully with dressing changes. The overall complication rate in this group was 19.2% (5/26). No surgical site infections occurred in either group. All complications in the OLIF-AF group occurred during the initial phase of technique implementation. There was no statistically significant difference in the early complication rates between the two groups (*p* > 0.05). The health cost after medical insurance payment in the MIS-TLIF group was less than that in the OLIF-AF group.

**Table 2:** Perioperative characteristics by type of procedure.

Variables	OLIF-AF (*n* = 31)	MIS-TLIF (*n* = 26)	*p*-value
Operative time (min)	125.7 ± 46.2	202.1 ± 66.4	<0.001*
Estimated blood loss (ml)	58.6 ± 30.5	143.5 ± 46.8	< 0.001*
Length of hospital stay (day)	8.6 ± 2.5	10.7 ± 3.5	0.009*
Perioperative complications	16.1% (5)	19.2% (5)	0.780
Nerve injury	3	2	
Dural sac tearing	0	2	
Incision poor healing	2	1	
Patients’ actual payment	31625.0 ± 2385.6	25859.4 ± 2100.8	< 0.001*

Patients’ actual payment is defined as the health costs after medical insurance payment, and the values are compared in Chinese Yuan (CNY). ∗ *p* < 0.05, the difference was significant.

### Radiological results

The imaging data of the two patient groups are compared in detail in Table 3. Baseline Comparability: Preoperatively, there were no statistically significant differences between the two groups in DH, ISA, CSA and SP (*p* > 0.05).

**Table 3:** Radiographic outcomes by type of procedure at 2-year follow-up.

Variables	OLIF-AF (*n* = 31)	MIS-TLIF (*n* = 26)	*p*-value
DH (mm)			
Preop (mean score)	8.5 ± 1.1	8.8 ± 1.3	0.409
Follow-up at 2 years	10.8 ± 1.4	9.8 ± 1.4	0.042*
ISA (°)			
Preop (mean score)	8.9 ± 2.6	8.9 ± 2.6	0.768
Follow-up at 2 years	12.9 ± 2.2	10.41 ± 3.1	0.015*
CSA (mm^2^)			
Preop (mean score)	71.5 ± 14.0	72.3 ± 13.9	0.532
Follow-up at 2 years	111.4 ± 13.5	101.2 ± 10.4	0.037*
SP (%)			
Preop (mean score)	17.4 ± 4.5	18.0 ± 5.2	0.712
Follow-up at 2 years	9.6 ± 1.8	9.4 ± 1.6	0.565
Fusion rate	93.5% (29)	92.3% (24)	0.855
Cage subsidence	3.2% (1)	3.8% (1)	0.505

∗ *p* < 0.05, the difference was significant.

At the 2-year follow-up, both groups showed significant improvements compared to preoperative baseline in DH, ISA, CSA and SP (*p* < 0.05). The OLIF-AF group demonstrated significantly greater improvement in DH, ISA, and CSA angle than the MIS-TLIF group, with statistically significant differences (*p* < 0.05). No statistically significant differences were observed in the SP, Bridwell fusion rate and cage subsidence rate between the two groups at the 2-year follow-up (*p* > 0.05). Throughout the follow-up duration, neither group experienced any long-term complications like intervertebral infection, implant loosening, or fracture.

### Clinical results

The follow-up results of the two groups of patients are presented in detail in Table 4. Baseline Comparability: Preoperatively, there were no statistically significant differences between the two groups in terms of low back pain VAS score, leg pain VAS score, or ODI score (*p* > 0.05).

**Table 4:** Comparison of postoperative VAS, ODI scores.

Scoring system	OLIF-AF (*n* = 31)	MIS-TLIF (*n* = 26)	*p*-value
VAS leg			
Preop (mean score)	5.7 ± 1.5	5.8 ± 1.7	0.958
Postop (1 week)	2.8 ± 1.4	2.6 ± 1.2	0.231
Follow-up at 3 months	1.5 ± 1.0	1.6 ± 1.0	0.745
Follow-up at 2 years	1.0 ± 0.4	1.1 ± 0.5	0.869
*p*-value (pre vs. post)	0.000	0.000	
VAS low back pain			
Preop (mean score)	5.4 ± 1.0	5.6 ± 1.4	0.427
Postop (1 week)	2.9 ± 0.7	4.0 ± 0.9	0.018*
Follow-up at 3 months	2.4 ± 1.4	3.5 ± 1.5	0.030*
Follow-up at 2 years	1.3 ± 0.8	1.3 ± 1.0	0.592
*p*-value (pre vs. post)	0.000	0.000	
ODI			
Preop (mean score)	57.6 ± 5.3	58.2 ± 6.4	0.080
Postop (1 week)	35.6 ± 7.3	42.0 ± 7.4	0.001*
Follow-up at 3 months	24.8 ± 3.4	21.1 ± 2.4	0.045*
Follow-up at 2 years	10.7 ± 2.4	10.7 ± 2.1	0.615
*p*-value (pre vs. post)	0.000	0.000	

VAS, Visual analog scale; ODI, Oswestry disability index; ∗ *p* < 0.05, the difference was significant.

**Leg Pain:** At 1 week, 3 months, and 2 years postoperatively, the leg pain VAS scores of both groups showed significant improvement compared with the preoperative baseline (*p* < 0.05), demonstrating a gradual decline over time. However, no statistically significant differences were observed between the two groups at any of the follow-up time points (*p* > 0.05).

**Low Back Pain and ODI:** At 1 week, 3 months, and 2 years after surgery, the low back pain VAS scores and ODI of both groups also improved significantly compared with the preoperative baseline (*p* < 0.05), likewise showing a progressive decrease over time. However, at the 1-week and 3-month follow-ups, the OLIF-AF group showed significantly better outcomes in low back pain VAS and ODI than the MIS-TLIF group (*p* < 0.05). By the 2-year follow-up, the intergroup differences in these two measures were no longer statistically significant (*p* > 0.05).

During follow-up, no patients in either group experienced worsened low back pain or radicular leg pain. There were no cases requiring revision surgery. In the MIS-TLIF group, two patients reported persistent low back distending pain at the 1-month follow-up, which improved within 3 months after oral nonsteroidal anti-inflammatory drug treatment and functional exercise.

## Discussion

A notable decline in functional status is associated with the restricted walking capacity caused by lumbar spinal stenosis secondary to DLS, which negatively impacts the quality of life (1). Historically, the mainstream surgical treatment for DLS has been decompression and fusion via a posterior approach, with MIS-TLIF being a standard technique. However, MIS-TLIF is often associated with complications such as neurological injury and residual low back pain (8). In recent years, minimally invasive lumbar interbody fusion techniques have advanced rapidly. These include anterior lumbar interbody fusion (ALIF), lateral lumbar interbody fusion (LLIF), extreme lateral interbody fusion (XLIF), and oblique lumbar interbody fusion (OLIF), among others (17). These approaches avoid the need for dissection through paraspinal muscles, resection of articular processes, or intrusion into the spinal canal and thecal sac. Specifically, OLIF accesses the anterolateral aspect of the lumbar spine through a natural anatomical plane, thereby minimizing injury to the back muscles and reducing harm to the psoas muscle (9). It has consequently become one of the most popular minimally invasive lumbar fusion techniques (16). Mechanistically, OLIF allows for the implantation of a larger cage into the disc space, which helps restore disc height, facilitate reduction of spondylolisthesis, and enlarge the area of the spinal canal (18). Furthermore, a larger cage provides a greater surface area for bone graft, which may lead to higher fusion rates (14).

However, conventional stand-alone OLIF carries risks of residual leg pain and cage displacement (11). Scholarly evidence indicates that supplemental internal fixation is often necessary for patients with DLS undergoing OLIF (12, 13). A finite element analysis comparing six types of supplemental fixation, including lateral single screw-rod fixation, suggested that supplemental posterior bilateral pedicle screw fixation provides the most stable biomechanical construct following OLIF (19). Nevertheless, this posterior augmentation diminishes the inherent minimally invasive advantages of OLIF and may lead to residual back pain (13). In contrast, utilizing a single-incision lateral approach with anterior single-screw-rod fixation (OLIF-AF) can enhance the stability of the construct without significantly increasing surgical complexity (20). The advantages of OLIF-AF over OLIF with posterior pedicle screw fixation include reduced total operative time—primarily by eliminating the need for patient repositioning, re-sterilization, re-draping, and a posterior approach—as well as decreased blood loss, avoidance of additional posterior soft tissue trauma, and potentially a shorter hospital stay (13).

In this study, we compared the clinical and radiographic outcomes of OLIF-AF and MIS-TLIF for treating neurogenic claudication caused by single-level low-grade DLS. The analysis showed that, compared with MIS-TLIF, OLIF-AF required fewer surgical steps, which translated into significantly shorter operating time, lower blood loss, and earlier discharge (14, 18). Both surgical methods improved patients’ low back pain VAS, leg pain VAS, and ODI scores. Patients who underwent OLIF-AF reported less low back pain—on the VAS—than the MIS-TLIF cohort at both 1 week and 3 months after surgery. This permits earlier mobilization for OLIF-AF patients. This is attributed to by avoiding the disruption of posterior spinal structures (e.g., paraspinal muscles, facet joints, laminae, and lumbar medial branch nerves) typical of posterior approaches, and by eliminating the implant-related irritation associated with posterior internal fixation, the OLIF-AF procedure results in milder early-stage back pain compared to MIS-TLIF. By the 2-year follow-up, the paraspinal muscles in the MIS-TLIF group had likely recovered, resulting in similar back pain scores between the groups. In this study, both techniques provided equivalent relief of leg pain at all postoperative time points. Whereas MIS-TLIF achieves direct decompression of neural structures, the OLIF technique utilizes a larger interbody cage to restore intervertebral height and reduce spondylolisthesis. This approach achieves decompression indirectly by expanding the cross-sectional area of the spinal canal and neural foramina, which relieves pressure on the cauda equina and nerve roots and results in the resolution of neurogenic claudication in the lower limbs (11, 13). However, it is noteworthy that leg pain in the OLIF-AF group was slightly higher (though not statistically significant) at 1 week postoperatively, possibly because OLIF provides indirect decompression, and the immediate nerve root decompression effect might be less pronounced than that of MIS-TLIF, which involves direct decompression (4). Furthermore, Zhao et al. suggested that cage subsidence within the first month after OLIF with anterior fixation might contribute to residual leg pain symptoms, which improve as interbody fusion occurs and stability increases by 3 months postoperatively (18); a similar trend was observed in this study. The ODI score was lower in the OLIF-AF group at 3 months but showed no significant difference between groups at the final follow-up. This might be because MIS-TLIF, utilizing posterior pedicle screw fixation, offers greater initial stability compared to the anterolateral vertebral body screw fixation used in OLIF-AF (17, 19).

We observed superior radiographic outcomes in the OLIF-AF group at the 2-year follow-up. OLIF-AF yielded markedly greater gains in disc height, intervertebral sagittal angle, and cross-sectional canal area than MIS-TLIF, findings that align with previous reports (14). Additionally, we found that there was no statistically significant difference in slip percentage between OLIF-AF and MIS-TLIF at the 2-year follow-up, indicating comparable long-term reduction effectiveness for low-grade degenerative spondylolisthesis. This is attributed to the larger cage size used in OLIF compared to MIS-TLIF, which aids in better restoration of lumbar anatomical alignment (21). Due to the larger contact area between the OLIF cage and the vertebral endplates, along with more graft material, the fusion efficacy of OLIF is theoretically superior to that of MIS-TLIF. However, at the 2-year follow-up, there was no statistically significant difference in interbody fusion rates between the two groups, consistent with previous reports (16). Cage subsidence is a common complication of lumbar interbody fusion surgery, it is commonly associated with endplate injuries and osteoporosis (12, 17). In our study, the cage subsidence rates in both the OLIF-AF and MIS-TLIF groups were lower than those reported in some previous literature (4), possibly related to careful preservation of the cortical endplate during disc space preparation and strict patient selection. It is crucial in OLIF to place the cage parallel to the disc space; otherwise, it may breach the endplate and lead to subsidence (21). Additionally, the insertion points for the anterior screws in OLIF-AF should be close to the adjacent endplates of the involved disc to provide good biomechanical support for interbody fusion and reduce the risk of cage subsidence (22). It should be noted that although the two groups of patients in this study differed in terms of bone graft fusion method, cage brand, and graft material, no significant differences were observed in the fusion rates and subsidence rates at the 2-year follow-up. We believe that the key to achieving consistent clinical outcomes lies in the following two aspects during surgery: first, thorough preparation of the intervertebral space to promote bony fusion; and second, preservation of the endplate to minimize the risk of cage subsidence and internal fixation failure.

No significant difference in complication rates was observed between the MIS-TLIF and OLIF-AF groups, either perioperatively or during the follow-up period. In OLIF-AF group, one patient developed temporary weakness of the iliopsoas (3.2%), and two patients had symptoms suggestive of sympathetic chain injury (6.4%). The numbers were within the normal range (4, 17, 20, 23). Hip flexion weakness, as reported by Johnson et al., is attributed to prolonged excessive retraction affecting the lumbar plexus and psoas muscle. Fortunately, these symptoms are generally transient (24). Postoperative sympathetic chain dysfunction (PSCD) is a well-recognized and not infrequent complication of oblique lumbar interbody fusion (OLIF), the findings of this study are consistent with the incidence rate observed in our earlier work, and the result showed that lumbar dextroscoliosis and psoas muscle location are risk factors for PSCD after OLIF (23). Preoperative attention to these factors is recommended. Additionally, intraoperative measures such as shortening the surgical duration and minimizing stretching of the lumbar sympathetic chain may help reduce the risk of PSCD. In our study, all affected patients in this study recovered in a short period. It should be noted that the results of this study incorporate the initial learning curve phase of our OLIF-AF technique implementation. Although early complications were relatively concentrated during the introductory period, this accurately reflects the natural evolution of the technique in real-world clinical practice. For most minimally invasive spine surgery techniques, successfully completing 25 consecutive cases allows surgeons to overcome the learning curve barriers, as evidenced by reductions in operative time and complication rates (25). Once beyond this learning phase, OLIF-AF demonstrates itself to be a safe and reliable technique.

Notably, when treating single-level low-grade degenerative spondylolisthesis with OLIF-AF, careful patient selection is crucial, avoiding those with osteoporosis, high BMI, or involved in heavy labor to reduce the risk of failure (12). Since OLIF is an indirect decompression procedure, it is not recommended for patients with bony spinal canal stenosis or obvious disc herniation (4).

### Limitations

This study has several limitations: 1. The cohort consisted of highly selected, retrospectively enrolled cases. Specific exclusions included procedures at the L5-S1 level and cases with pre-existing facet joint fusion. Furthermore, the surgical approach was not randomized. Notably, for cases where the anatomical plane between the psoas major and abdominal aorta was indistinct, MIS-TLIF was preferentially selected, which may have introduced a source of selection bias. Therefore, prospective, double-blind, randomized controlled trials are warranted to mitigate the potential impact of this bias on the results. 2. The current mid-term follow-up data are insufficient to fully evaluate the long-term stability of clinical outcomes; extended observation periods are needed to obtain more comprehensive prognostic data. 3. Limited by the single-institution data source and sample size, the generalizability (external validity) of the results may be affected. Future in-depth analysis involving multi-center, large-sample cohorts is necessary to fully demonstrate the efficacy advantages and differential characteristics of the two surgical techniques.

## Conclusion

Although both OLIF-AF and MIS-TLIF achieve satisfactory clinical and radiographic outcomes in treating neurogenic claudication caused by single-level low-grade degenerative lumbar spondylolisthesis, OLIF combined with anterior single-screw-rod fixation offers advantages over MIS-TLIF in terms of reduced surgical trauma, faster recovery, and better early clinical and radiographic results, while demonstrating equivalent long-term efficacy.

## Data Availability

The original contributions presented in the study are included in the article/supplementary material, further inquiries can be directed to the corresponding author.
